# Crude Garden Cress Seed Oil (*Lepidium sativum* Linn.) Enhances Post-Thawed Boar Sperm Quality

**DOI:** 10.3390/ani14223178

**Published:** 2024-11-06

**Authors:** Vassakorn Khophloiklang, Panida Chanapiwat, Kampon Kaeoket

**Affiliations:** 1 Semen Laboratory, Department of Clinical Sciences and Public Health, Faculty of Veterinary Science, Mahidol University, Phutthamonthon 73170, Thailand; vassakorn.khp@student.mahidol.edu (V.K.); panida.chn@mahidol.edu (P.C.); 2 Faculty of Veterinary Science, Rajamangala University of Technology Srivijaya, Thung Yai 80240, Thailand

**Keywords:** amino acids, antioxidant, boar sperm, fatty acids, garden cress oil

## Abstract

This study aimed to examine the effects of crude garden cress seed oil (CGCSO) on frozen–thawed boar sperm qualities. Semen ejaculates (*n* = 12) were collected and further divided into six equal aliquots based on CGCSO concentrations (0, 0.5, 1, 1.5, 2, and 2.5% *v*/*v*) in the freezing extender. Semen samples were processed and cryopreserved utilizing the traditional liquid nitrogen vapor technique. Subsequently, semen samples were thawed in a thermos with warm water at 50 °C for 12 s and evaluated for sperm morphology using scanning electron microscopy, sperm motility using a CASA, sperm viability, acrosome integrity, mitochondrial function, MDA level, total antioxidant capacity (TAC), glutathione peroxidase (GSH-Px), and catalase (CAT) activity. The results indicated that 1% CGCSO resulted in superior post-thaw sperm characteristics, including enhanced sperm morphology, motility, viability, acrosome integrity, and mitochondrial function. Particularly, the total motile sperm increased by 16.5%, progressive motile sperm increased by 13.0%, viability improved by 15.1%, acrosome integrity increased by 14%, and mitochondrial function improved by 14.1% compared to the control group. CGCSO treatment at 1% and 1.5% exhibited the lowest level of MDA (45.73 ± 11.2 and 45.73 ± 11.3 µmol/L, respectively) compared to the other groups. The CGCSO-supplemented groups showed higher values of TAC, GSH-Px, and CAT than the control group but not significantly.

## Simple Summary

Boar sperm demonstrate significant sensitivity to the conditions during cryopreservation, with various factors, and show a reduction in their fertilization capacity due to damaging effects on sperm cell membranes. We investigated the effects of crude garden cress seed oil (CGCSO) on sperm viability, motility, acrosome integrity, mitochondrial function, and antioxidant activity in frozen boar semen. CGCSO improved post-thawed semen qualities such as the total motility, progressive motility, viability, acrosome integrity, and mitochondrial function as well as reduced lipid peroxidation. As a result, we suggested that 1% CGCSO in the freezing extender diminished the oxidative damage caused by cryopreservation and led to improved sperm quality after thawing.

## 1. Introduction

Artificial insemination (AI) technology had been utilized to improve livestock breeding, intensely for industrial-scale and specialized productions. The advancements in in vitro storage and long-distance transportation have greatly promoted herd breeding by accelerating the cross-country exchange of animal genetics through semen cryopreservation technology. Nevertheless, the sperm membrane structures experience partially irreversible damage due to the unbalanced production or accumulation of reactive oxygen species (ROS) during extended storage periods and freezing–thawing cycles, resulting in physical, chemical, and oxidative damage. Aberrant membrane permeability and DNA strand breakage decrease sperm motility and fertility capacity [1].

However, boar sperm exhibit a high level of sensitivity to the conditions experienced during cryopreservation, with multiple factors influencing their response. It is crucial to emphasize that the frozen boar semen leads to a significant reduction in fertilization capacity due to detrimental effects on spermatozoa membranes [2]. This is important since polyunsaturated fatty acids (PUFAs) are abundant in mammalian sperm plasma membranes [3]. Additionally, the composition of lipids in the plasma membrane of sperm is crucial in determining its characteristics related to movement, sensitivity to low temperatures, ability to survive, and the integrity of its membrane [4]. It is worth mentioning that PUFAs are highly enriched in their boar sperm’s plasma membrane compared to other species [3]. Fatty acids (FAs) have a crucial role in shaping the structure and function of sperm within their membranes. They facilitate important membrane fusion events that occur during fertilization [5]. PUFAs are notable among these FAs due to their capacity to penetrate the membrane of sperm cells, enhance the pliability of the sperm plasma membrane, maintain its structural and functional integrity, strengthen the resistance of the acrosome membrane to osmotic stress, and provide protection against physiological or thermal variations encountered during cryopreservation [6]. However, reducing the amount of PUFAs in the sperm plasma membrane can cause oxidative stress, indicating a relationship between lipid peroxidation and sperm motility, viability, and morphology [7]. In order to protect sperm cells from the detrimental effects of free radicals, a strong combination of antioxidants and FAs has been used in the process of semen freezing. Earlier research has demonstrated that bioactive peptides from palm kernel meal protein hydrolysate [8] and animal protein [9], curcumin [10], and resveratrol [11] have also been used to make frozen boar semen for a better post-thawing quality. During the past decades, fatty acids derived from different sources, including DHA in egg yolks [12], olive oil [13], and camellia oil [14], have shown their positive effect on the fatty acids existing in the sperm plasma membrane. This, in turn, leads to an improvement in the quality of boar semen after thawing. In addition, adding fatty acids into the freezing extender has been shown to improve parameters such as total motility, progressive motility, viability, and decreased lipid peroxidation [15].

Crude garden cress seed oil (CGCSO) is a multifunctional oil derived from the seeds of *Lepidium sativum* Linn., which belongs to the Brassicaceae family. This species is native to southwest Asia and has been mentioned in Europe and Asia for centuries [16]. Garden cress seed extract has been found to possess antimicrobial, antihypertensive, antioxidant, antispasmodic, antidiarrheal, anti-asthmatic, hypoglycemic, and hypolipidemic properties, as supported by many studies [17,18]. Garden cress seeds contain approximately 20% oil and are a rich source of fatty acids such as linolenic acid (omega 3) and oleic acid (omega 6) [19]. CGCSO is regarded as a relatively stable oil due to the presence of natural antioxidants, such as tocopherol, phytosterol, and carotenoids, which help prevent the oil from becoming rancid [19]. These components may increase sperm qualities and decrease the peroxidation of lipid during the cryopreservation of boar semen. Nevertheless, there is a lack of knowledge about the impact of CGCSO on the quality of boar semen cryopreservation. This study was aimed to clarify the antioxidant effect of CGCSO by assessing the qualities of post-thawed boar sperm.

## 2. Materials and Methods

### 2.1. Animal

Twelve semen ejaculates were collected during January to February 2024 from twelve boars of different breeds (four Landrace, four Large White, and four Duroc) aged between 1.5 and 3 years old. The sample size of twelve boars was determined using the G-Power software version 3.1. The calculation utilized the sperm viability data of frozen–thawed boar semen obtained from Thongrueang et al. [20]. The boars were housed individually in the pen of a commercial farm located in Nakhon Pathom province, Thailand. All the boars had free access to chlorine-treated water through automated watering systems and feeding with in-house feed. Their daily feed intake was similar and regulated to meet the requirements for semen production, which totaled 3 kg per day. These boars were routinely used to collect semen for artificial insemination.

### 2.2. Chemicals and Extenders

**Figure 1:**
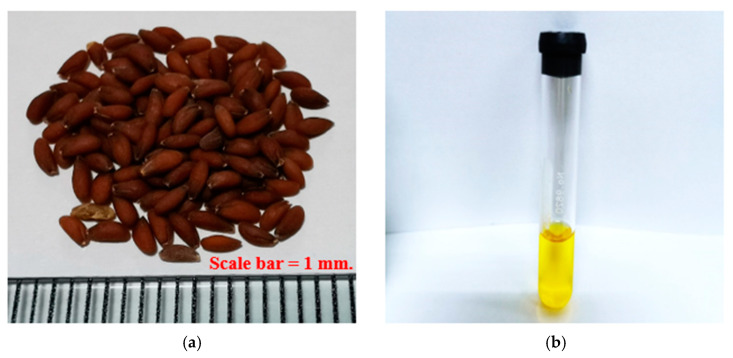
(**a**) Garden cress seed (*Lepidium sativum* Linn.) before oil pressing. (**b**) Garden cress seed oil (CGCSO) pressed by oil-pressing machine.

In this study, the crude garden cress seed oil was extracted using a cold pressing method (Figure 1) (Oil Press Machine Oil Extractor for Cold/Hot Squeeze, Dezhou, China). Three different extenders were utilized for the cryopreservation of boar semen, which are as follows: Firstly, the commercial semen extender was Optim-I.A. (Magapor, Zaragoza, Spain). The second extender consisted of a 20% egg yolk and 11% lactose solution with varying concentrations of CGCSO (0%, 0.5%, 1%, 1.5%, 2%, and 2.5% *v*/*v*) [14]. Each portion was diluted with or without (control) the CGCSO substance using an equal volume of extender; and the third extender consisted of 89.5% second extender, with 9% (*v*/*v*) glycerol and 1.5% (*v*/*v*) Equex-STM^®^ (Nova Chemical Sales Inc., Scituate, MA, USA) [11].

### 2.3. Fatty Acid Determination

The fatty acid profiles of CGCSO were determined using the methodology outlined in the Association of Official Analytical Chemists 2005 (AOAC) [12]. The total fat content of 32 fatty acids was determined by calculating the area under the peak and expressed as the amount of each fatty acid per 100 g of total fatty acid [14].

### 2.4. Amino Acid Determination

The amino acid profiles of garden cress seeds were determined using high-performance liquid chromatography, following an in-house method TE-CH-372, which was derived from the Official Journal of the European Communities L257 [21] and TE-CH-373 based on the Journal of Food Chemistry [22].The amino acid profiles were analyzed by the Central Laboratory (Thailand) Co., Ltd., Bangkok, Thailand.

### 2.5. Polyphenol Determination

The total phenolic content (TPC) of garden cress seed extract was measured using a Folin–Ciocalteu spectrophotometric method with certain alterations. In summary, a total of 0.5 mL of crude seed extract was combined with 2.5 mL of 10-fold-diluted Folin–Ciocalteu’s phenol reagent. The mixture was then left to react for a duration of 5 min. Next, 2 mL of a 7.5% Na_2_CO_3_ solution was introduced, and the total volume was adjusted to 10 mL using deionized water. The absorbance at 760 nm was determined after 1 h reaction at room temperature. The measurement was compared to a standard curve of a prepared solution of gallic acid (GA), and the total phenolic content was expressed as mg of gallic acid equivalents (GAE) per gram of dry weight (mg of GAE/g of dw) [23].

### 2.6. Assessment of Total Antioxidant Activity (Seed and Oil)

The 2,2-diphenyl-1-picrylhydrazyl (DPPH) radical scavenging assay was used to evaluate the garden cress seeds. Following a gradual dilution of the crude extracts with water, a volume of 10 µL from the dilution was transferred into a 96-well plate and 185 µL of DPPH solution in a 50% ethanol was added into each well. The plate was then agitated at room temperature for 5 min. The alteration in light absorption at a wavelength of 550 nm was subsequently quantified using a Multilabel Counter. The radical scavenging activity of the sample is measured in terms of epigallocatechin gallate (EGCG) equivalent per gram of the sample (µmol/g). This measurement was based on the concentration required to inhibit 50% of the red coloration at 550 nm [24].

The lipophilic antioxidant assay, oxygen radical absorbance capacity (ORAC), was used to assess the CGCSO. The CGCSO was dissolved in 250 µL of acetone and subsequently diluted with 750 µL of a 7% RMCD (randomly methylated â-cyclodextrin) solution (50% acetone/50% water, *v*/*v*). A 7% RMCD solution was employed for any further dilution. The Trolox standards were dissolved in a 7% RMCD solution used for the lipophilic assay. To perform lipophilic analysis, 40 µL of the solution was added to the 48-well microplate. The fluorescein solution was added to the microplate reader, followed by the addition of AAPH (2,2′-azobis(2-amidino-propane) dihydrochloride; 17.2 mg/mL, 9.4 µmol/well) in a volume of 150 µL. The readings were commenced promptly [25].

### 2.7. Semen Collection and Preparation

Semen samples were collected using the glove-hand technique [26]. Each semen sample was then thoroughly examined using different criteria, such as semen volume, sperm movement, sperm concentration, sperm viability, and the presence of intact sperm cells (sperm morphology was assessed using William’s staining method). Only the semen samples with a minimum motility of 70% and morphology of 80% were chosen for the cryopreservation procedure [8].

### 2.8. Semen Freezing and Thawing Process

Every individual sperm sample was cryopreserved using a traditional liquid nitrogen method. After collection, the semen was diluted with the OPTIM Optim-I.A. extender in a 1:1 volume ratio. The diluted semen was subsequently transferred into 50 mL centrifuge tubes and equilibrated at 15 °C for 120 min. Following the equilibration period, the samples were centrifuged at 15 °C with a force of 800× *g* for 10 min (LMC-4200R, Biosan, Riga, Latvia). After centrifugation, the supernatant was removed, and the sperm pellet was mixed with a second extender at a concentration of 1.5 × 10^9^ sperm/mL, using a volume ratio of approximately 1–2:1. In this step, the sample was further divided into six groups, each with different CGCSO concentrations (0, 0.5, 1, 1.5, 2, and 2.5% *v*/*v*). Next, the samples were cooled to 5 °C for 90 min. Afterward, each set of samples was combined with a third extender to reach a concentration of 1.0 × 10^9^ sperm/mL and yield final CGCSO concentrations of 0, 0.48, 0.97, 1.45, 1.93, and 2.41% *v*/*v*, respectively. The mixture was then placed into 0.5 mL straws (IMV Technologies, L’Aigle, Basse-Normandie, France). The semen straws were cryopreserved by exposing them to nitrogen vapor at 4 cm above the liquid nitrogen level for 20 min, resulting in a cooling rate of −20 °C/min. The straws were then rapidly immersed in liquid nitrogen [8] and stored in a tank at −196 °C for further analysis. Prior to sperm assessments, the cryopreserved semen was held for 12 h, and then the frozen samples were heated to 50 °C for 12 s and diluted (1:6 volume ratio) with a warmed Optim-I.A. extender in water bath at 37 °C for 15 min [8].

### 2.9. Assessment of Sperm Motility

Sperm motility assessments were implemented using a computer-assisted sperm motility analysis (CASA) with an AndroVision^®^ system (Minitube, Tiefenbach, Germany). Concisely, a volume of 3 µL of the semen sample was carefully moved into a disposable counting chamber (Leja^®^ 20 µM, IMV Technologies, L’Aigle, Basse-Normandie, France) and kept at a consistent temperature of 37 °C throughout the analysis. Each analysis required the counting of at least 600 sperm cells, obtained by examining five distinct fields in each sample. The results were presented as percentages, indicating the overall movement of sperm, the forward movement of sperm, and different aspects of sperm movement, including curvilinear velocity (VCL, µm/s), average pathway velocity (VAP, µm/s), straight-line velocity (VSL, µm/s), amplitude of lateral head displacement (ALH, µm), wobble (WOB, %), straightness (STR, %), and linearity (LIN, %). Sperm that are capable of movement were characterized by a VCL of at least 24 µm/s and an ALH of more than 1 µm. Progressive motility (PMOT) is defined as having a VCL of at least 48 µm/s and a VSL of less than 10 µm/s. The total motility (MOT) is calculated by adding up the different subpopulations of sperm motility, which are classified based on specific thresholds for curvilinear velocity (VCL). These subpopulations include local motility (VCL ≥ 24 and <48 µm/s), slow motility (VCL ≥ 48 and <80 µm/s), and fast motility (VCL ≥ 80 µm/s) [8].

### 2.10. Assessment of Sperm Viability

The viability of sperm was assessed using a staining method involving SYBR-14 (L7011(A); Live/Dead™ Sperm viability kit, Invitrogen, Waltham, MA, USA) and Ethidiumhomodimer-1 (EthD-1, E1169, Invitrogen, Waltham, MA, USA). To summarize, 50 µL of semen was combined with 2.7 µL of a 0.54 µM SYBR-14 solution in DMSO, resulting in a final concentration of 0.27 µM. Additionally, 10 µL of a 4.68 µM EthD-1 solution in PBS was added and incubated at 37 °C for 15 min. A fluorescence microscope with a magnification of 400× was used to assess 200 sperm. The sperm were categorized into two groups: viable and non-viable sperm. Nuclei from viable sperm with intact plasma membranes fluoresced green, while those from dead sperm or sperm with damaged plasma membranes fluoresced red. The proportion of viable and non-viable sperm was calculated [8].

### 2.11. Assessment of Acrosome Integrity

**Figure 2:**
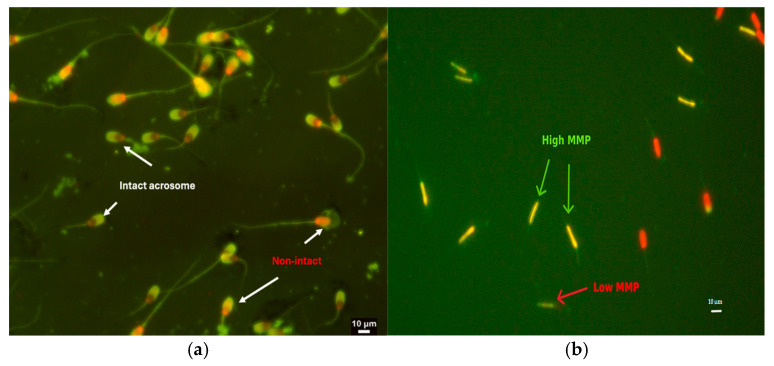
Post-thawed boar sperm parameters using specific fluorescent dyes (400×). (**a**) Intact and non-intact acrosomes were stained using FITC-PNA/EthD-1 staining. (**b**) Sperm with high and low mitochondrial membrane potential (MMP) using JC-1/PI staining.

The integrity of the acrosome was evaluated using the fluorescein isothiocyanate-labeled peanut agglutinin (FITC-PNA; L7381, Sigma-Aldrich Co., Darmstadt, Germany) and EthD-1 staining protocol. Briefly, 10 µL of diluted semen was mixed with an equal volume of 4.68 µM EthD-1 solution in PBS and incubated at 37 °C for 15 min. Subsequently, a 5 µL aliquot was applied to a glass slide and allowed to dry. The sample was then fixed with 95% ethanol for 30 s and air-dried. Forty µL of FITC-PNA (100 µg/mL in PBS) were evenly spread across the slides. The slides were subsequently placed in a humid chamber at 4 °C for 30 min. The slides were then washed with cold PBS and air-dried once again. Using a fluorescence microscope with a magnification of 1000×, a total of 200 live sperm were analyzed. The sperm with intact acrosomes fluoresced green with a smooth contour on the acrosomal area, whereas the sperm with damaged acrosomes fluoresced green with an irregular contour (Figure 2). The results were expressed as the percentage of live sperm with intact acrosomes [26].

### 2.12. Assessment of Mitochondrial Membrane Potential

The mitochondrial membrane potential was assessed using a staining method with the following fluorescent dyes: 5, 5′, 6, 6′-tetrachloro-1, 1′, 3, 3′, -tetraethylbenzimidazolylcarbocyanine (JC-1, T3168, Invitrogen, Waltham, MA, USA), and propidium iodide (PI, L7011(B); Live/Dead™ Sperm viability kit, Invitrogen, Waltham, MA, USA). Fifty µL of diluted semen was mixed with 3 µL of a 2.4 mM PI solution and 3 µL of a 1.53 mM JC-1 solution in DMSO, then incubated at 37 °C for 10 min, with a final concentration of 129 µM PI and 82 µM JC-1. The total 200 live sperm (PI-negative) were examined under a fluorescence microscope at a magnification of 400×. The sperm with a high mitochondrial membrane potential fluoresced yellow–orange at the midpiece, whereas those with a low membrane potential fluoresced green (Figure 2). The percentage of viable sperm with a high mitochondrial membrane potential was calculated [8].

### 2.13. Assessment of Lipid Peroxidation

The assessment of lipid peroxidation was conducted by quantifying the level of malondialdehyde (MDA) using a colorimetric lipid peroxidation assay kit (ab118970, Abcam^®^, Cambridge, UK). For sample preparation, a volume of 250 µL of post-thawed semen in each treatment was centrifuged at 20,000× *g* for 10 min. The supernatant was collected for further analysis. Following instructions, the supernatant sample (200 µL) was mixed with 600 µL of thiobarbituric acid (TBA) solution and incubated at 95 °C for 60 min to produce an MDA–TBA adduct, which was then measured. The MDA–TBA product was measured at OD_532_ using a microplate reader (SPECTROstar Nano, BMG LABTECH, Ortenberg, Germany). A 2 mM MDA standard was prepared and diluted in a series for the colorimetric assay’s standard curve. The MDA levels were determined using an MDA standard curve and reported as µmol/L [8].

### 2.14. Assessment of Total Antioxidant Capacity

Total antioxidant capacity (TAC) was assessed using a colorimetric assay kit (ab65329, Abcam^®^, Cambridge, UK) according to the manufacturer’s instructions. In brief, 250 µL of post-thawed semen in each treatment was centrifuged at 20,000× *g* for 10 min, and the supernatant was collected and diluted (1:100) in double-distilled water. The 100 µL of diluted samples and standards were added to the 96 well plate. All standards and samples were mixed with 100 µL of Cu^2+^ solution and incubated at room temperature for 90 min. Following incubation, the absorbance was promptly measured at OD_450_ using a microplate reader (SPECTROstar Nano, BMG LABTECH, Ortenberg, Germany). A 1 mM Trolox standard was prepared and diluted in a series for the colorimetric assay’s standard curve. The TAC was determined using the TAC standard curve and calculated as µmol/L [14].

### 2.15. Assessment of Glutathione Peroxidase Activity

Glutathione peroxidase (GSH-Px) activity was assessed by using a colorimetric lipid peroxidation assay kit (ab102530, Abcam^®^, Cambridge, UK). The assay is based on the principle that GSH-Px catalyzes cumene hydroperoxide’s oxidation of GSH. In the presence of glutathione reductase and NADPH, the oxidized glutathione (GSSG) was converted into a reduced form with the concomitant oxidation of NADPH. Before the analysis, the frozen semen sample was prepared according to the previous study [27]. A 50 µL sample was aliquoted into a 96-well microtiter plate and processed following the manufacturer’s protocol. The decrease in absorbance was measured at OD_340_ using a microplate reader (SPECTROstar Nano, BMG LABTECH, Ortenberg, Germany). A 1 mM NADPH standard was prepared and serially diluted for the standard curve. The amount of NADPH was determined, and GSH-Px activity was calculated as mU/mL.

### 2.16. Assessment of Catalase Activity

Catalase (CAT) activity was evaluated using the commercial kit (STA-341 OxiSelect™ Catalase Activity Assay Kit, Cell Biolabs, Inc., San Diego, CA, USA). The principle of assay is based on catalase’s reaction to decompose H_2_O_2_. Before the analysis, the frozen semen sample was prepared according to the previous study [27]. The 20 µL of samples and standards were transferred to a 96-well microtiter plate and processed according to the manufacturer’s instruction. The absorbance was measured at OD_520_ using a microplate reader (SPECTROstar Nano, BMG LABTECH, Ortenberg, Germany). The catalase standard solution (10,000 units/mL) was diluted to prepare a series of the catalase standard curve. CAT activity was calculated as U/mL.

### 2.17. Evaluation of Sperm Morphology by Scanning Electron Microscopy (SEM)

The semen samples were initially fixed in a 2.5% glutaraldehyde solution in PBS at 4 °C for 24 h (Electron Microscopy Sciences, Hatfield, MA, USA). Following fixation, the samples were rinsed three times with PBS for 15 min each to remove excess fixative. The semen samples were post-fixed with 0.1% osmium tetroxide in PBS (Sigma-Aldrich, Munich, Germany) at room temperature for 1 h before being washed three times with PBS for 15 min each. The fixed samples were dehydrated through a graded ethanol series (70%, 80%, 90%, 95%, and 100%), each for 15 min. The sperm cells were mounted on an SEM stub and coated with 50 nm platinum particles [28]. SEM imaging was performed using a scanning electron microscope (JEOL, JSM-IT500LA, Akishima, Japan). The detailed morphological features of sperm cells were observed [14].

### 2.18. Statistical Analysis

The statistical analysis was conducted using IBM SPSS Statistics for Windows, version 26.0 (SPSS Inc., Chicago, IL, USA). The normality of the data was tested using a Shapiro–Wilk test. The sperm parameters, specifically progressive motility and VAP, had a non-normal distribution. As a result, a Log10 transformation was applied to these parameters. The parameters, such as total motility, progressive motility, sperm motility patterns, sperm viability, acrosome integrity, mitochondrial membrane potential, MDA levels, and total antioxidant capacity, were reported as the mean ± standard error of the mean (SEM). Levene’s test was used to assess the homogeneity of variances, and a one-way ANOVA was conducted to compare the means. The sperm parameters among treatment groups were compared using Duncan’s multiple-range test. A *p*-value < 0.05 indicated a statistically significant difference.

## 3. Results

### 3.1. Fatty Acid Determination

Among the 32 fatty acids profiles analyzed, linolenic acid (omega 3, 6), oleic acid (omega 9), and arachidic acid were the most prominent in the composition of fatty acids in CGCSO, as shown in Table 1. The highest proportion of fatty acid determination was PUFAs (i.e., 42.19 g/100 g), followed by MUFAs and SFAs (i.e., 31.50 and 26.66 g/100 g, respectively).

### 3.2. Amino Acid Determination

Amino acid compositions of garden cress seed are shown in Table 2. Results revealed that garden cress seed contained 9 essential amino acids (histidine, isoleucine, leucine, lysine, methionine, phenylalanine, threonine, tryptophan, and valine) and 9 non-essential amino acids (alanine, arginine, aspartic acid, cysteine, glutamic acid, glycine, proline, serine, tyrosine), while hydroxylysine and hydroxyproline were not found in the garden cress seed.

### 3.3. In Vitro Antioxidant Activity of Seed and CGCSO Determination

For scavenging activity, the electron transfer (ET) ability of the seed to the free-radical DPPH was determined. The seed exhibited a DPPH value of 4046.96 mmol TE/100 g, while the hydrogen atom transfer (HAT) ability of CGCSO was shown as an ORAC value of 2628 µmol TE/100 mL. The polyphenol content in garden cress seed was found to be 1963.90 mg GA/100 g, as indicated in Table 3.

### 3.4. Effects of CGCSO on Sperm Motility

**Figure 3:**
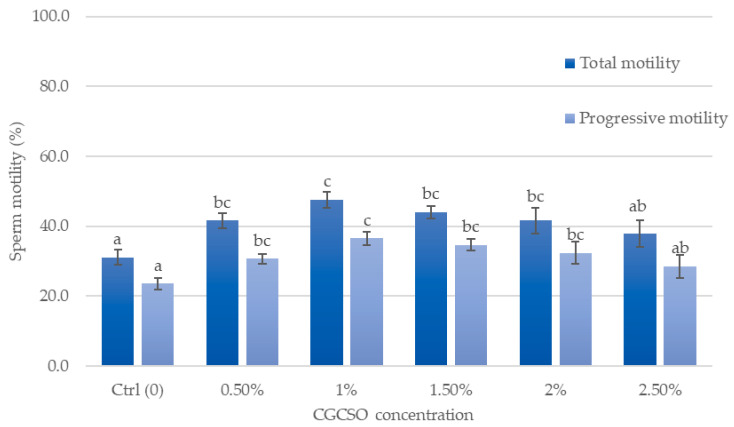
The effect of CGCSO on total motility and progressive motility. The bars in the graph represent the mean ± SEM. Different letters indicate a statistically significant difference (*p* < 0.05).

Table 4 presents descriptive statistics of fresh boar semen quality. All measurements reveal that the quality of the fresh semen samples meets the acceptable standards. Figure 3 depicts the influence of CGCSO on sperm motility. The results suggest that adding 1.0% CGCSO to the semen extender resulted in a higher sperm quality after thawing than other concentrations. The addition of 1.0% CGCSO to semen samples increased total motility and progressive motility by 16.5% and 13.0%, respectively, compared to the control group (47.5 ± 2.4% vs. 31.1 ± 2.1% and 36.5 ± 1.6% vs. 23.5 ± 1.6%, respectively). Moreover, the 1.0% CGCSO group showed a considerably higher percentage of sperm movement characteristics (VCL, VSL, VAP, ALH, WOB, STR, and LIN) compared to the control group (*p* < 0.05) (Table 5).

### 3.5. Effects of CGCSO on Sperm Viability

**Figure 4:**
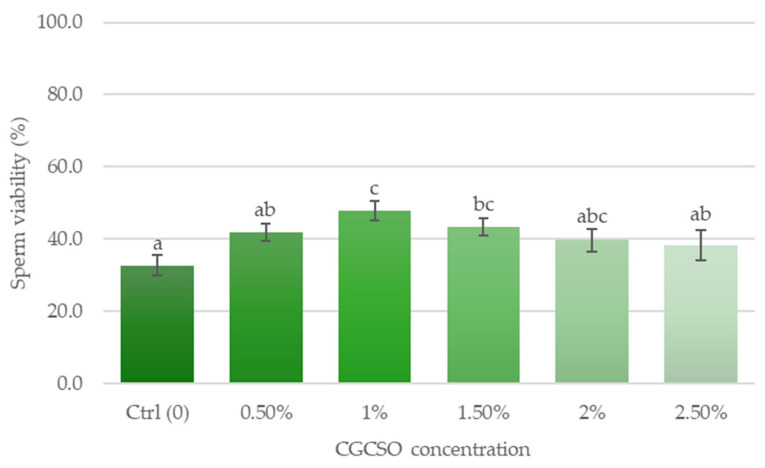
The effect of CGCSO on sperm viability. The bars in the graph represent the mean ± SEM. Different letters indicate a statistically significant difference (*p* < 0.05).

Sperm viability was higher in the 1% CGCSO and 1.5% CGCSO groups (i.e., 47.8 ± 2.8% and 43.3 ± 2.5%, respectively) compared to the other groups (Figure 4). However, the viability of sperm did not differ significantly between the 1% CGCSO and 1.5% CGCSO groups. The control group demonstrated the lowest sperm viability (32.7 ± 2.9%).

### 3.6. Effects of CGCSO on Sperm Acrosome Integrity

**Figure 5:**
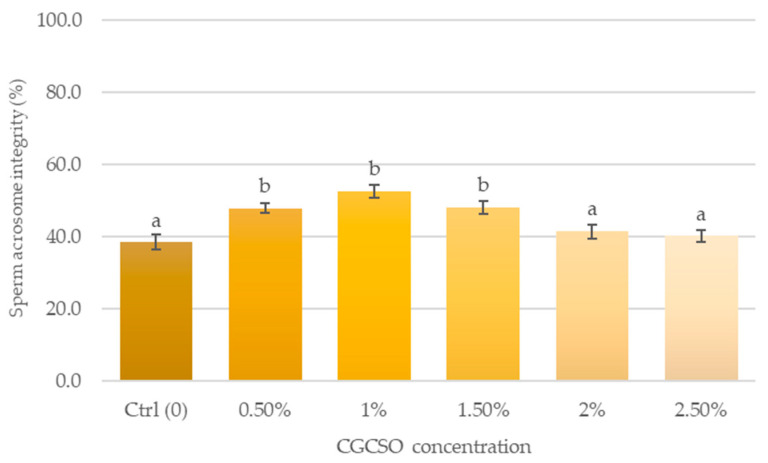
The effect of CGCSO on sperm acrosome integrity. The bars in the graph represent the mean ± SEM. Different letters indicate a statistically significant difference (*p* < 0.05).

Differences in the integrity of the acrosome were noted between the control and treatment groups (Figure 5). Nevertheless, the 1.0% CGCSO group exhibited the greatest percentage of acrosome integrity (52.5 ± 1.7%), which was significantly 14.0% higher than the control group.

### 3.7. Effects of CGCSO on Mitochondrial Membrane Potential

**Figure 6:**
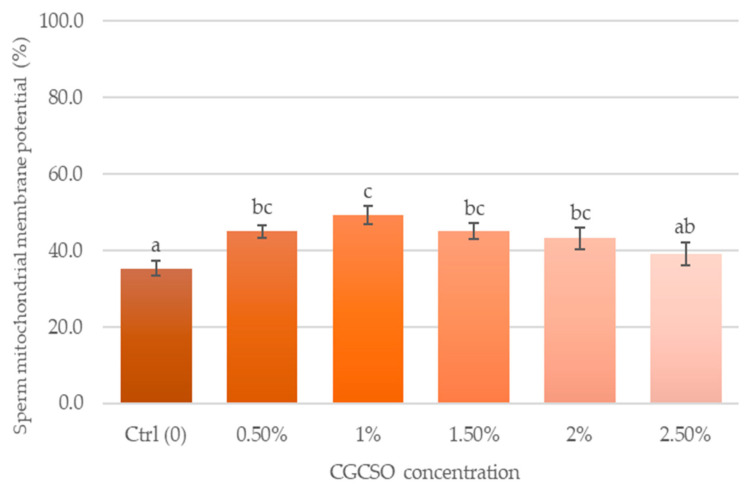
The effect of CGCSO on sperm mitochondrial membrane potential. The bars in the graph represent the mean ± SEM. Different letters indicate a statistically significant difference (*p* < 0.05).

Figure 6 displays the findings of mitochondrial membrane potential. The group supplemented with 1.0% CGCSO showed a significantly higher percentage of mitochondrial function (49.3 ± 2.4%) compared to the control group, with an increase of 14.1% (*p* < 0.05). The lowest mitochondrial membrane potential was found in control group (35.3 ± 1.9%).

### 3.8. Effects of CGCSO on Lipid Peroxidation

**Figure 7:**
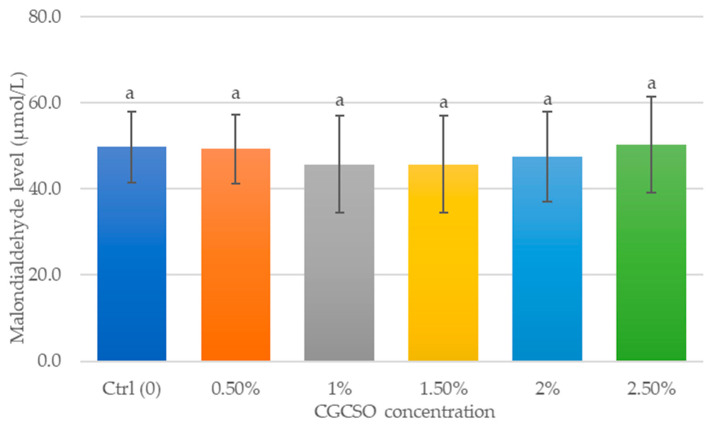
The effect of CGCSO on the lipid peroxidation (MDA levels). The bars in the graph represent the mean ± SEM. Different letters indicate a statistically significant difference (*p* < 0.05).

Figure 7 depicts the impact of CGCSO on the process of lipid peroxidation during cryopreservation. The results showed a decrease in MDA levels in the groups supplemented with 1.0% and 1.5% compared to the control group. However, this difference was not statistically significant (*p* = 0.7).

### 3.9. Effects of CGCSO on Total Antioxidant Capacity

**Figure 8:**
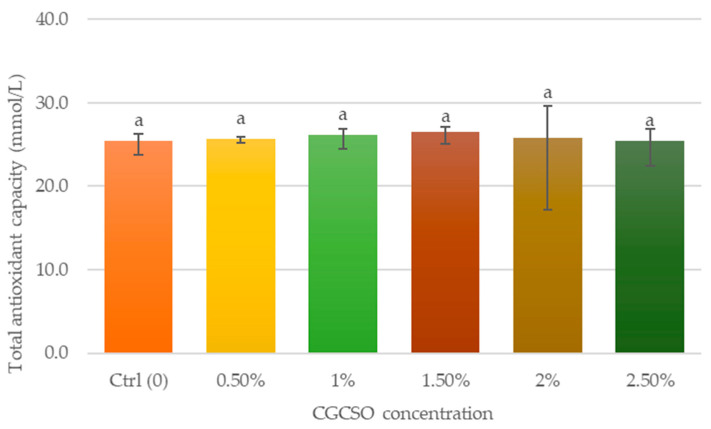
The effect of CGCSO on the total antioxidant capacity (TAC). The bars in the graph represent the mean ± SEM. Different letters indicate a statistically significant difference (*p* < 0.05).

There was no statistical significance between the treatment groups and the control group (Figure 8). Nevertheless, there was a noticeable inclination towards higher total antioxidant capacity (TAC) in the treatment groups as compared to the control group (*p* = 0.7).

### 3.10. Effects of CGCSO on Sperm GSH-Px and CAT Activity

As is shown in Table 6, GSH-Px and CAT activity in the CGCSO-treated groups was higher than that in the control group. The highest GSH-Px activity of cryopreserved boar sperm was observed in the group supplemented with 2.0%, and there was a clear tendency towards increased sperm CAT activity in the treatment groups compared to the control group. However, this difference was not statistically significant (*p* > 0.05).

### 3.11. Effects of CGCSO on Sperm Morphology

**Figure 9:**
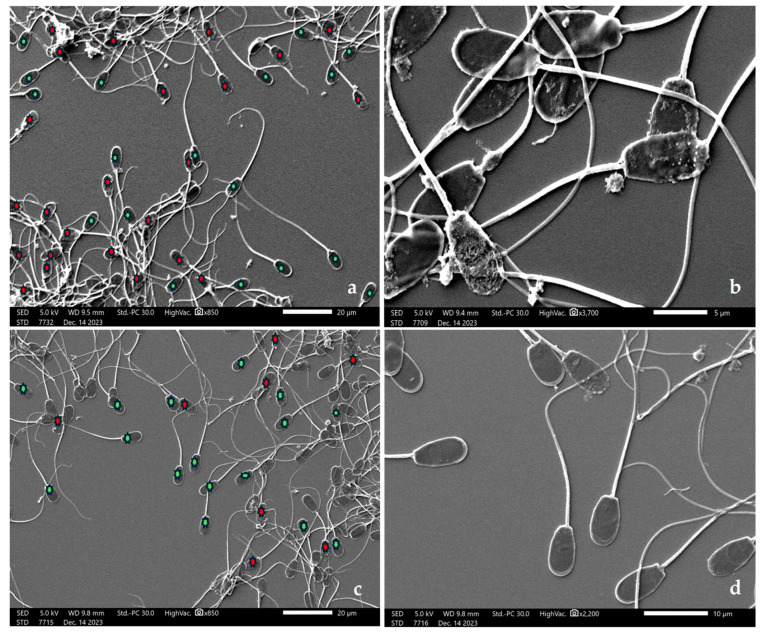
Scanning electron micrographs of post-thawed boar sperm. The control group: ((**a**), 850×) showed a high number of non-intact sperm morphology; ((**b**), 3700×) showed plasma membrane and acrosome damage. The 1% CGCSO group: ((**c**), 850×) showed a high number of intact sperm morphology (intact plasma membrane and acrosome); ((**d**), 2200×) showed intact sperm morphology. The sperm (**a**,**c**) with intact morphology were labeled with a green asterisk, while the sperm with non-intact morphology were labeled with a red asterisk.

The imaging of the sperm morphology using SEM is displayed in Figure 9. The control group exhibited a higher non-intact sperm morphology, including damage to the plasma membrane and acrosome (Figure 9a,b). In contrast, the 1% CGCSO group displayed higher number of intact sperm morphology and a lower degree of abnormal morphology compared to the control group (Figure 9c,d).

## 4. Discussion

Previous research on the cryopreservation of boar sperm has demonstrated that these sperm cells are highly susceptible to freezing–thawing techniques, resulting in oxidative stress and an excessive production of ROS. As a result, the antioxidant system becomes imbalanced, as it is unable to effectively counteract the excessive presence of ROS [29]. As a result, this disparity significantly influences the properties of semen, affecting sperm motility, mitochondrial activity, membrane permeability, and overall sperm functions, thereby reducing their effectiveness in artificial insemination programs [1,29].

The initial findings of the CGCSO report indicated that incorporating CGCSO into the freezing extender has an interesting positive effect on various parameters of post-thawed boar semen. The parameters encompass enhanced sperm morphology as shown by SEM, total motility, progressive motility, viability, acrosome integrity, mitochondrial membrane potential, and kinetic motility parameters including VSL, VAP, WOB, STR, and LIN. In addition, the current findings demonstrated that the treatment groups had superior levels of total antioxidant capacity, glutathione peroxidase activity, catalase activity, and lower levels of MDA than the control group, especially when 1% of CGCSO was added. This effect can be elucidated by the fact that sperm cells absorbed and utilized fatty acids and amino acids from the freezing extender in order to counteract ROS which were produced during cryopreservation [29]. Consequently, this process led to a decrease in lipid peroxidation of the sperm plasma membrane and internal organelles in treatment groups [30]. Nevertheless, the wide range of values observed in the parameters of this study may be due to individual differences among boars, including variations in sperm biochemistry, composition of seminal plasma, physiological factors, genetic variations in the testis and epididymis, and the presence of specific fatty acids, as previously mentioned [4]. Considering all the results together, it is suggested that 1% CGCSO is the most effective concentration for cryopreserving boar semen. The present results also indicated that fatty acids, amino acids, and phenolic compounds, by nature of their antioxidant characteristics, protect sperm cells during the freezing process, thereby reducing the damage to their membrane, acrosome, and mitochondria. Our study confirms previous findings that fatty acids such as oleic and linoleic acid [31] or oleic acid and palmitic acid [32] have a positive effect on the motility, viability, acrosome reaction, mitochondrial membrane potential, and ATP production of chilled boar sperm. In accordance with the study in poultry, the supplementation of oleic acid into a chilled extender resulted in improved rooster semen quality, reduced MDA levels, and increased TAC [33]. Besides those fatty acids, it has been demonstrated that the presence of omegas 3, 6, 9, and DHA in freezing extender enhanced the quality of boar and buffalo semen after thawing, particularly the plasma membrane and acrosome integrity [26,34]. This study discovered that CGCSO, which is abundant in omega 3 and 9 fatty acids (i.e., oleic and linoleic acid), contributed to maintaining membrane fluidity and exhibited antioxidant properties by scavenging free-radicals and upregulating antioxidant enzymes, subsequently, preventing sperm from cryodamage during the frozen–thawed process [30]. In terms of the polyphenol and amino acid content, CGCSO not only contained substantial levels of polyphenols but also amino acids, including glutamic acid, aspartic acid, arginine, and others. In agreement with the present results, the inclusion of the polyphenol extract from garden cress seed in the feed supplement led to improved sperm movement, viability, concentration, reproductive performance, reproductive hormone levels, milk yield, serum antioxidant status, and a decrease in abnormal sperm morphology in rabbit [35,36].

Besides fatty acids, the seed oil of *Lepidium sativum* Linn. contains a variety of amino acids, including essential amino acids such as histidine, isoleucine, leucine, lysine, methionine, phenylalanine, threonine, tryptophan, and valine. Non-essential amino acids are glutamic acid, aspartic acid, arginine, glycine, proline, alanine, serine, tyrosine, and cystine [37,38]. A study found that certain amino acids, such as arginine, histidine, alanine, valine, isoleucine, and leucine, found in palm kernel meal protein hydrolysate, significantly enhanced the quality of post-thawed boar sperm [8]. Other studies also reported that cysteine [7], proline [39], lysine [40], and bioactive peptides could improve the quality of post-thawed boar semen and ram semen [9]. The addition of amino acids such as arginine, lysine, methionine, tryptophan, valine, and threonine to the feed as a feed additive had a beneficial effect on fresh and chilled boar semen quality [41,42,43]. It has also been documented that amino acids provide multiple functions, such as decreasing free radicals, protecting cells from denaturation, and providing an oxidizable substrate to sperm [44]. In order to elucidate the roles of amino acids in cryopreservation, Alanine, glycine, glutamine, histidine, and proline have been employed as cryoprotectant agents in many species due to their ability to inhibit lipid peroxidation or regulate osmotic mechanisms [45,46,47]. The proline supplemented in freezing extender has also been reported to enhance sperm motility and protects against free-radical-induced damage by stabilizing the structure and function of the cell membrane during the freezing process in vitro [48]. Additionally, alanine and glutamine also improved the motility and viability of post-thawed semen in ram, bull, and stallion [47,49,50]. Glutamine is recognized for its regulatory function in several cell-specific processes, including metabolism, cellular integrity, protein synthesis, and protein degradation [51]. Aside from the benefit to the sperm mechanism as mentioned above, amino acids in CGCSO, such as arginine and histidine, consist of positively charged amino acid groups [52]. The amino acid groups mentioned have the ability to transfer protons or electrons to free-radical molecules. Additionally, hydrophobic amino acids including alanine, valine, isoleucine, and leucine, with their enhancing hydrophobicity, can lead to higher lipid solubility and consequently increase their antioxidative activity [52]. All these demonstrated mechanisms may explain the superior results in motility, viability, intact acrosomes, and mitochondrial activity observed in the CGCSO-treated groups.

However, in the present study, it is worth noting that an excessively high concentration of CGCSO exhibited a slightly negative effect on sperm quality. This phenomenon has also been observed with other antioxidants [8,14]. An excess of antioxidants may disturb the equilibrium between free radicals and antioxidants in mammalian cells. Excess antioxidants may bind to other substances, thereby diminishing redox signaling and gene expression. This disruption can subsequently lead to a reduction in antioxidant capacity and ATP synthesis in mitochondria [53].

The garden cress seed and its oil in this study were constituents of polyphenols, omega 3, 6, and 9 fatty acids, as well as essential and non-essential amino acids, which is in agreement with previous reports. The fatty acids present in the seed showed considerable variation, including oleic acid (18–30%), α-linoleic acid (7.5–30%), α-linolenic acid (26–34%), and arachidic acid (2–3.5%). The antioxidant activity of both the seed and its oil can be assessed using chemical methods based on different mechanisms by which plant antioxidants exert their effects [54,55]. This study utilized total polyphenol content, DPPH (a hydrogen atom transfer-based method), and ORAC (a single electron transfer-based method) assays to assess the antioxidant activity of garden cress seed and its oil. The findings revealed high levels of polyphenol and ORAC activity in the oil as well as substantial DPPH activity in the seed (i.e., 1963.90 mg eq GA/100 g, 2628 µmoles TE/100 mL and 4046.96 mmoles TE/100 g, respectively). These results indicated that both the seed and its oil, with their rich polyphenol content, possess significant antioxidant capabilities by participating in free-radical chain reactions, which greatly enhance the antioxidant activity of the oil [56]. In the present study, the CGCSO antioxidant activity showed a higher ORAC level (2628 µmoles TE/100 mL) than the level in crude rice bran oil (i.e., 920 µmoles TE/100 mL) [57], in which the latter oil could improve motility, membrane integrity, and acrosome integrity of post-thawed boar semen in a previous study [26]. The antioxidant properties of garden cress seed oil and seed extract have been extensively demonstrated through various assays, including 2,2′-azino-bis (3-ethylbenzthiazoline-6-sulphonic acid) radical-scavenging (ABTS), ferric reducing antioxidant power (FRAP), total radical-trapping antioxidant parameter (TRAP) assays, DPPH, reducing power (RP), and β-carotene/linoleic acid assays [38]. These assays have shown that the oil and seed extracts exhibit strong antioxidant activity. In addition, it has been shown that seed oil contains the natural antioxidant tocopherols, including α-tocopherol, γ-tocopherol, and δ-tocopherol, as well as various phenolic compounds like flavonoids, quercetin, caffeic acid, chlorogenic acid, and gallic acid [38]. These phenolic compounds, particularly caffeic acid, chlorogenic acid, gallic acid, and quercetin, have been used to improve post-thawed boar semen quality [58]. The CGCSO in the present study exhibited a high level of polyphenols, which could significantly impact the post-thawed boar semen quality in the treatment group.

The current findings demonstrate that adding a 1% of CGCSO to the freezing extender significantly enhanced the quality of post-thawing boar semen. On the other hand, concentrations exceeding 1.5% resulted in a decline in the quality of post-thawing boar semen. Despite potential benefits, CGCSO, as a natural substance, poses challenges in terms of standardization when used as an additive in freezing boar semen extenders. It is worth noting that fatty acid composition of each batch of CGCSO should be analyzed before using it in any specific experiment to ensure consistency and effectiveness. Furthermore, field fertility tests using this frozen–thawed boar sperm for artificial insemination in pig farms is needed in order to examine its fertilizing capacity.

## 5. Conclusions

Based on the overall findings, it can be inferred that CGCSO, due to its richness in fatty acids, amino acids, and polyphenols, effectively reduced the production of ROS during cryopreservation. In frozen–thawed boar sperm, the decrease in ROS helped to prevent lipid peroxidation and improved sperm qualities, such as sperm morphology, motility, viability, intact acrosomes, mitochondrial membrane potential, and total antioxidant capacity. Adding CGCSO at a concentration of 1% to the freezing extender greatly improved the quality of frozen–thawed boar semen, whereas a detrimental effect was found at concentrations greater than 1.5%.

## Figures and Tables

**Table 1:** Fatty acids composition of CGCSO.

Type of Fatty Acid	g/100 g of Total Fatty Acid
C12:0 (Lauric Acid) (Saturated fatty acids)	0.01
C14:0 (Myristic Acid) (Saturated fatty acids)	0.09
C16:0 (Palmitic Acid) (Saturated fatty acids)	9.12
C18:0 (Stearic Acid) (Saturated fatty acids)	3.24
C18:1n9c (Oleic Acid) (MUFA) (Omega 9)	21.00
C18:2n6c (Linoleic Acid) (PUFA) (Omega 6)	10.74
C18:3n3 (Linolenic Acid) (PUFA) (Omega 3)	29.83
C18:3n6 (Gamma Linolenic Acid) (PUFA) (Omega 6)	0.09
C20:0 (Arachidic Acid) (Saturated fatty acids)	12.51
C20:1 (Eicosenoic acid) (MUFA)	4.07
C20:2 (Eicosadienoic acid) (PUFA)	0.49
C20:3n3 (cis-11,14,17-Eicosatrienoic acid) (PUFA) (Omega 3)	0.65
C22:0 (Docosanoic acid) (Saturated fatty acids)	1.06
C22:1n9 (Erucic acid) (MUFA) (Omega 9)	5.58
C22:2 (cis-13,16-Docosadienoic acid) (PUFA)	0.05
C23:0 (Tricosanoic acid) (Saturated fatty acids)	0.03
C24:0 (Tetracosanoic acid) (Saturated fatty acids)	0.59
C24:1 (Nervonic acid) (MUFA)	0.83
Overall	
Saturated fatty acids	26.66
Monounsaturated fatty acids	31.50
Polyunsaturated fatty acids	42.19
Omega 3 fatty acids	30.47
Omega 6 fatty acids	10.83
Omega 9 fatty acids	30.66
Omega 6/omega 3 fatty acids	0.40

**Table 2:** Amino acids composition of garden cress seed.

Amino Acid Profiles	mg/100 g
Glutamic acid	3654.67
Aspartic acid	2015.52
Arginine	1544.63
Leucine	1543.73
Glycine	1339.58
Lysine	1252.68
Valine	1221.74
Proline	1099.73
Phenylalanine	1088.55
Alanine	1002.76
Serine	970.13
Isoleucine	953.19
Threonine	917.73
Histidine	606.28
Tyrosine	553.45
Methionine	295.92
Tryptophan	264.54
Cystine	<200.00
Hydroxylysine	ND
Hydroxyproline	ND

ND: Not detected.

**Table 3:** The antioxidant profiles of garden cress seed and oil.

Measurement	Results
Seed	
Total antioxidant activity of ET (DPPH) (mmol TE/100 g)	4046.96
Total polyphenol (mg eq GA/100 g)	1963.90
Oil	
Total antioxidant activity of HAT (ORAC) (µmol TE/100 mL)	2628.00

**Table 4:** Sperm quality of fresh boar semen (*n* = 12).

Parameters *	Mean ± SEM	Range
Concentration (×10^6^ sperm/mL)	309.7 ± 21.0	171–390
Total motility (%)	90.7 ± 1.3	95.4–83.6
Progressive motility (%)	83.2 ± 2.7	92.7–68.7
Sperm viability (%)	93.7 ± 0.8	97.6–90.2
Acrosome integrity (%)	93.9 ± 0.9	97.0–89.4

* = Results are expressed as mean ± SEM (*n* = 12).

**Table 5:** Effect of CGCSO on sperm motility patterns of post-thawed boar sperm.

Parameters *			Concentrations (% *v*/*v*)			
	Ctrl (0)	0.50	1	1.50	2	2.50
VCL	36.7 ± 2.4 ^ab^	40.4 ± 2.2 ^ab^	45.3 ± 2.7 ^a^	44.3 ± 3.1 ^ab^	39.2 ± 4.9 ^ab^	36.0 ± 4.0 ^b^
VSL	11.4 ± 1.2 ^a^	14.1 ± 0.7 ^ab^	17.5 ± 1.1 ^b^	14.9 ± 1.0 ^ab^	14.0 ± 2.1 ^ab^	12.8 ± 1.6 ^a^
VAP	15.9 ± 1.2 ^a^	18.4 ± 1.0 ^ab^	21.9 ± 1.5 ^b^	20.3 ± 1.5 ^ab^	18.0 ± 2.5 ^ab^	16.6 ± 2.1 ^a^
ALH	0.48 ± 0.02 ^a^	0.50 ± 0.02 ^a^	0.53 ± 0.02 ^a^	0.54 ± 0.03 ^a^	0.48 ± 0.04 ^a^	0.45 ± 0.03 ^a^
WOB	43.1 ± 0.6 ^a^	45.4 ± 0.6 ^ab^	47.7 ± 1.0 ^b^	45.6 ± 0.7 ^ab^	44.9 ± 1.2 ^a^	45.4 ± 1.0 ^ab^
STR	68.3 ± 2.4 ^a^	77.1 ± 1.1 ^bc^	79.8 ± 1.1 ^c^	74.4 ± 1.5 ^c^	76.7 ± 1.5 ^bc^	77.3 ± 0.9 ^bc^
LIN	29.3 ± 1.3 ^a^	35.2 ± 0.9 ^b^	38.7 ± 1.0 ^c^	33.9 ± 0.9 ^b^	34.9 ± 1.4 ^b^	35.2 ± 1.0 ^b^

* = Results are expressed as mean ± SEM (*n* = 12). ^a,b,c^ Means with different superscripts in the same row are significantly different between groups (*p* < 0.05), VCL (µm/s): curvilinear velocity, VSL (µm/s): velocity straight line, VAP (µm/s): average pathway velocity, ALH (µm): amplitude of lateral head displacement, WOB (%): wobble, STR (%): straightness, LIN (%): Linearity.

**Table 6:** Effect of CGCSO on the antioxidant status of post-thawed boar sperm.

Parameters *			Concentrations (% *v*/*v*)			
	Ctrl (0)	0.50	1	1.50	2	2.50
GSH-Px (mU/mL)	38.1 ± 15.7 ^a^	56.7 ± 24.1 ^a^	67.9 ± 19.1 ^a^	56.5 ± 24.2 ^a^	93.4 ± 15.2 ^a^	90.4 ± 10.3 ^a^
CAT (U/mL)	0.90 ± 0.06 ^a^	0.98 ± 0.04 ^a^	0.97 ± 0.06 ^a^	0.94 ± 0.04 ^a^	1.11 ± 0.08 ^a^	1.08 ± 0.07 ^a^

* = Results are expressed as mean ± SEM (*n* = 5). Means with different superscripts in the same row are significantly different between groups (*p* < 0.05).

## Data Availability

No new data were created or analyzed in this study. Data sharing is not applicable to this article.
